# Extracting information from free-text electronic patient records to identify practice-based evidence of the performance of coronary stents

**DOI:** 10.1371/journal.pone.0182889

**Published:** 2017-08-11

**Authors:** Yoon Seob Kim, Dukyong Yoon, JungHyun Byun, Hojun Park, Ahram Lee, Il Hyun Kim, Sukhoon Lee, Hong-Seok Lim, Rae Woong Park

**Affiliations:** 1 Department of Biomedical Informatics, Ajou University School of Medicine, Suwon, Gyeonggi-do, Republic of Korea; 2 Department of Biomedical Sciences, Ajou University Graduate School of Medicine, Suwon, Gyeonggi-do, Republic of Korea; 3 Department of Software Convergence Engineering, Kunsan National University, Gunsan Jeollabuk-do, Republic of Korea; 4 Department of Cardiology, Ajou University School of Medicine, Suwon, Gyeonggi-do, Republic of Korea; Nagoya University, JAPAN

## Abstract

**Background and objective:**

Percutaneous coronary intervention (PCI) using drug-eluting stents (DES) is an indispensable treatment for coronary artery disease. However, to evaluate the performance of various types of stents for PCI, numerous resources are required. We extracted clinical information from free-text records and, using practice-based evidence, compared the efficacy of various DES.

**Materials and methods:**

We developed a text mining tool based on regular expression and applied it to PCI reports stored in the electronic health records (EHRs) of Ajou University Hospital from 2010–2014. The PCI data were extracted from EHRs with a sensitivity of 0.996, a specificity of 1.000, and an F-measure of 0.995 when compared with a sample of 200 reports. Using these data, we compared the performance of stents by Kaplan-Meier analysis and the Cox hazard proportional regression.

**Results:**

In the self-validation analysis comparing the first-generation to the second-generation DES, the second-generation DES was superior to the first-generation DES (hazard ratio [HR]: 0.423, 95% confidence interval [CI]: 0.284–0.630) in terms of target vessel revascularization (TVR), showing similar findings to the established results of previous studies. Among the second-generation DES, the biodegradable-polymer DES tended to be superior, with a risk of TVR (HR: 0.568, 95% CI: 0.281–1.147) falling below than that for the durable-polymer DES approximately 1 year after the index procedure. The Endeavor stent had the highest TVR risk among the newer generation DES (HR: 2.576, 95% CI: 1.273–5.210).

**Conclusions:**

In this study, we demonstrated how to construct a PCI data warehouse of PCI-related parameters obtained from free-text electronic records with high accuracy for use in the post surveillance of coronary stents in a time- and cost effective manner. Post surveillance of the practice based evidence in the PCI data warehouse indicated that the biodegradable-polymer DES might have a lower risk of TVR than the durable-polymer DES.

## Introduction

Percutaneous coronary intervention (PCI) is an indispensable method for the treatment of coronary artery disease [1]. In 2010, it was estimated that 492,000 patients underwent PCI procedures in the United States, of which approximately 454,000 were PCI procedures using stents [2]. Because of the high incidence of PCI and stent use, even a small difference in the performance of stents could result in an enormous difference in patient outcome. With the rapid development of stent technology aimed at lowering the rate of stent failure, not enough is known about the outcomes of new products. Therefore, constant and rapid surveillance of the performance of emerging stents, and comparisons among various types of stents, is required, but doing so remains difficult.

The earliest form of coronary angioplasty involved only balloon catheters, while coronary stents were first introduced in 1989 to prevent restenosis after angioplasty by maintaining lumen integrity [1, 3]. However, in-stent restenosis and the need for frequent revascularization arose as bare metal stents (BMS) began to be used [4]. The first-generation drug eluting stents (DES), which interfere with cell proliferation, were thus developed, further lowering the risk of restenosis [5]. However, these stents were shown to increase the risk of late stent thrombosis and delay arterial healing due to their interfering with the cellular environment [6, 7]. Moreover, the structure of the first-generation Sirolimus-eluting stents was also vulnerable to mechanical complications such as stent fracture [8–10]. Subsequently, second-generation DES were designed to decrease the risk of late target lesion revascularization, improving the reliability of DES [11, 12]. Biodegradable polymer-coated stents were developed to improve long-term outcome by providing temporary function and stepping away of the polymers [13]. It has been shown that biodegradable polymers improve the safety and efficacy of second-generation stents compared to first-generation DES [14–16]. However, further studies are needed to address the differences between the newer generation durable polymer DES (DP-DES) and biodegradable polymer DES (BP-DES) [17].

After clinical adoption of the first-generation DES (which had shortcomings), technological advances have included improvements in materials and design, the employed polymer, and the eluted drugs, rendering adverse clinical events very rare. Therefore, it is very important, but has become increasingly difficult, to evaluate and compare performance among stents [17]. A decrease in the incidence of major adverse cardiac events (MACE) has made it difficult to compare the performance of various stents with clear statistical validity. Also, techniques for revascularization are undergoing rapid evolution, which render many studies obsolete or difficult to interpret [18]. This problem derives mainly from the fact that traditional research methods, such as prospective cohort studies, require vast resources. This limitation warrants a new research method that carries less economic burden and is less time-consuming.

Thus, a retrospective cohort study using information from electronic health records (EHRs) was considered, as records that have been originally stored for practice purposes can also be used for research. However, using EHRs poses a barrier as coronary angiography (CAG) reports are written in free-text; free-text files cannot be directly analyzed using existing tools, and in the present study, CAG reports were too numerous to review manually.

We overcame this obstacle by developing a method of analysis using regular expression. Regular expression refers to a formal programming language for specifying strings, and is used practically to identify particular strings in texts [19]. Using this method, we extracted variables related to PCI from CAG reports originally stored in EHRs. Patient characteristics, such as age, sex, and a history of diabetes mellitus and hypertension, were also processed from the EHRs. The data were used to build a PCI data warehouse for statistical analyses.

Using free-text CAG reports from the Ajou University Hospital between February 2010 and October 2014, we extracted information and conducted a retrospective cohort study of a real-world population that had undergone PCI. To validate our data, we examined and compared the clinical performance of different stents and analyzed the hazard ratios (HR) of target vessel revascularization (TVR) between first- and second-generation DES. Following validation of our program, we explored the efficacy and safety of BP-DES versus DP-DES with the risk of TVR as the primary endpoint.

## Materials and methods

### Ethics statement

This study was approved and informed consent was waived by the Institutional Review Board of Ajou University Hospital because the anonymized data was analyzed retrospectively.

### Data source

Patients who underwent PCI procedures were identified from the EHRs of Ajou University Hospital. A total of 13,567 coronary reports (CAG reports with or without descriptions of PCI procedures performed after diagnostic CAG) written between February 2010 and October 2014 were analyzed by regular expression. Patient characteristics including clinical and demographic information, drug prescriptions, and diagnoses were also extracted from EHRs.

### Data processing of coronary angiography reports

We extracted information from the free-text PCI reports and loaded it into a data warehouse; we applied regular expression using the R programming language to complete this task. The ‘stringr’ package developed by Hadley Wickham was used primarily for extracting patterns [20].

A table of pre-defined vessel and stent terms was created, taking advantage of the fact that PCI reports had recurring structures around these words (e.g., newline, colon, slash). The terms in their presumed locations were extracted, manually reviewed by a cardiologist, and categorized (Table 1). The various forms of the terms on the list were then used for extraction.

**Table 1:** Vessel and stent terms used in PCI reports and their categories in this study.

	Vessel/stent categories			Vessel/stent terms used in PCI reports
Vesssel	LAD			LAD, Di, D1, D2, diagonal/Dx
	LCx			LCx, OM, RI/Ramus
	LM			LM, LM-LAD, LM-LCx
	RCA			RCA, PDA, PLV, PLB
Stent	BMS			Vision, Genoss, Coroflex Blue, Zeta
	DES	First generation		Cypher, Coroflex Please, Taxus
		Second generation	Durable polymer	Resolute family, Promus family, Xience family, Endeavor
			Biodegradable polymer	Biomatrix, Desyne, Nobori, Orsiro

LAD, left anterior descending artery; LCx, left circumflex; LM, left main; RCA, right coronary artery; BMS, bare metal stent; DES, drug-eluting stent; D1, the first diagonal artery; D2, the second diagonal artery; Dx, diagonal artery; OM, obtuse marginal; PDA, posterior descending artery; PLV, posterior left ventricular branch; PLB, posterior lateral branch.

**Fig 1:**
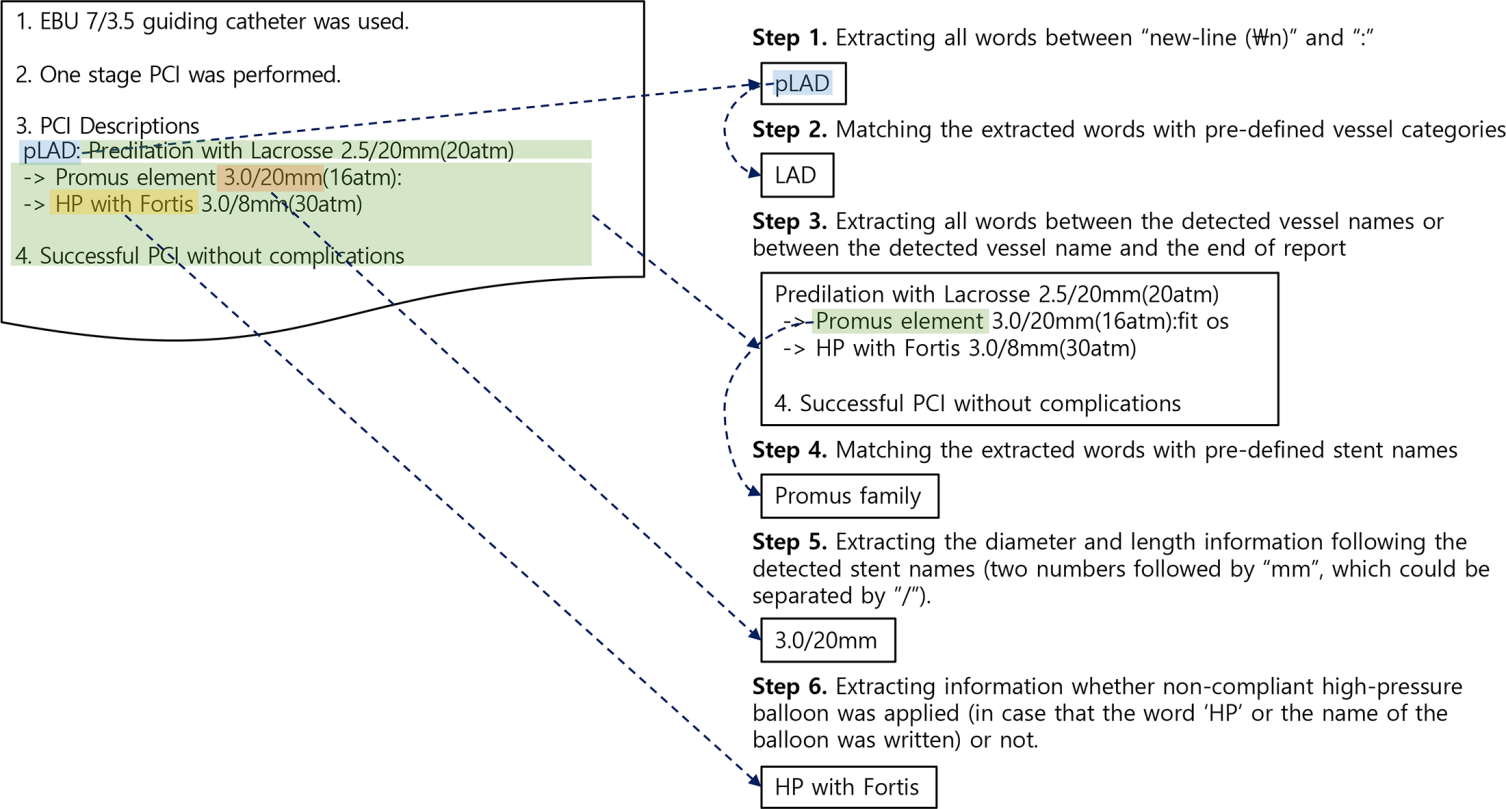
The percutaneous coronary intervention (PCI)-related parameter information extraction process from free-text PCI procedure reports.

The overall process is shown in Fig 1. First, we extracted all words between newline (\n) and colon (Step 1). These words mainly referred to the target vessels in the procedure. We then compared the extracted words with the pre-defined vessel categories shown in Table 1 (Step 2). After identifying the target vessels, we went on to find the names, diameters, and lengths of the stents, and ascertained whether a non-compliant adjuvant balloon was applied (Step 3). The stent names were extracted by matching them with pre-defined stent names (Step 4), and the diameters and lengths of the stents were captured by extracting two numbers followed by “mm”, which were separated by “/” (Step 5). If any of these three variables (stent name, diameter, and length) were missing, it was determined that there was no record of inserting a stent in the corresponding vessel. Finally, we identified whether a non-compliant high-pressure balloon was applied by checking whether “HP” or the name of a non-compliant high-pressure balloon was written in the report (Step 6). Script samples and a CAG report are shown in S1 File.

The scripts for data extraction were first developed by three individual programmers. Upon completion, their results were compared side-by-side, and the scripts underwent two cycles of revision for higher conformity. Subsequently, the matching parts of the three tables were used for the PCI data warehouse. The parts that did not reach consensus were filled in through manual review of the original reports.

The completed PCI data warehouse itself was validated by manual review. To evaluate the degree of agreement among the three different programmers and between information retrieved by each script and the data filled out manually from 200 randomly chosen samples (the gold standard), we compared the list of pairs that consisted of the target vessel and the stent categories inserted into the target vessel. The kappa values for the primary, secondary, and tertiary pairwise comparisons of the extracted results between the three programmers were 0.659, 0.974, and 0.986, respectively. As shown in Table 2, the mean script sensitivities, specificities, and F-measures approached those of the gold standard after two cycles of revision. All of the comparisons were performed after all revisions were complete, eliminating the possibility that the revisions were biased.

**Table 2:** Mean sensitivities, specificities, and F-measures for each iterative round.

Round	Mean Sensitivity	Mean Specificity	Mean F-measure
Primary	0.741	0.999	0.864
Secondary	0.960	1.000	0.959
Tertiary	0.991	1.000	0.991

### Study covariates and outcome variables

The outcome variable for this study was TVR within 2 years after index PCI with stent implantation. TVR was defined as cases where patients had repeated PCI procedures on a target vessel.

The patient characteristic covariates included age, sex, hypertension, type 2 diabetes mellitus, and the duration of dual antiplatelet therapy (DAPT). The procedural characteristic covariates included the total length and average diameter of the implanted stent(s) in each target vessel, the approach site (radial artery or femoral artery), and whether the PCI was emergent. Patients with type 2 diabetes were extracted according to the process defined by Kho et al. [21]. Hypertension was defined as either the assignment of the ICD-10 diagnostic code I10 or prescription of at least one type of antihypertensive drug.

### Statistical analysis

First, an analysis for data warehouse validation was performed to confirm that the PCI data warehouse reflected established real-world findings. We compared the risk of TVR between first- and second-generation DES using Kaplan-Meier analysis and the log-rank test, and all study covariates were adjusted using the Cox proportional hazard regression model. We used the PCI data warehouse to analyze the differences between DP-DES and BP-DES using the same statistical methods as described above. Within-class comparisons of stent groups were also performed. The types of stents that were used in greater than 100 procedures, as determined from the EHRs, were selected and categorized, including the Promus family, the Xience family, the Resolute family, the Endeavor, the Nobori, and the Biomatrix stents. Prior to each analysis, cases where a vessel was treated with more than one category of stents were excluded (i.e., vessels that were treated with both BP-DES and DP-DES). All statistical analyses were performed using the R package (R Development Core Team, Vienna, Austria). A *p*-value < 0.05 was considered significant.

## Results

### Data processing

The final consensus version of the data extraction program by three different programmers, including a manual review of elements for which consensus was not attained (220 of 5,612 descriptions, 3.9%), was used to generate the PCI data warehouse. A negative predictive value of 1.000 and a positive predictive value of 0.995 were observed when these data were compared with the gold standard data of the target vessel and stent categories. Sensitivity was 0.996, specificity was 1.000, and the F-measure was 0.995, indicating that the data warehouse can be used in research settings.

### PCI data warehouse construction

A PCI data warehouse containing information from PCI reports was constructed, and included information from 5,612 target vessels of 3,817 patients who underwent index PCI between 2010 and 2014. The patient and procedural characteristics of the index procedures are shown in Table 3.

**Table 3:** Baseline, procedural, and outcome findings.

Variable	All	DP-DES	BP-DES	*p*-value*
No. of patients, n	3817	2468	568	-
Age, mean ± SD	62.75 ± 11.77	62.19 ± 11.80	63.14 ± 11.61	0.080
Male, n (%)	2724 (71%)	1799 (73%)	386 (68%)	0.021
Hypertension, n (%)	1756 (46%)	1126 (46%)	263 (46%)	0.806
Diabetes mellitus, n (%)	746 (20%)	489 (20%)	116 (20%)	0.788
Average duration of DAPT (days), mean ± SD	572.59 ± 444.77	559.29 ± 425.71	549.12 ± 384.32	0.578
Duration of DAPT over 12 months, n (%)	2326 (61%)	1510 (61%)	347 (61%)	1.000
No. of treated vessels, n (%)				
LAD	2565 (67%)	1474 (60%)	349 (61%)	0.480
RCA	1346 (35%)	806 (33%)	156 (27%)	0.019
LCx	1288 (34%)	639 (26%)	145 (26%)	0.900
LM	413 (11%)	312 (13%)	37 (7%)	< 0.001
Average diameter of stents per target vessel (mm), mean ± SD	2.61 ± 1.19	3.11 ± 1.19	3.07 ± 1.19	0.020
Total length of stents per target vessel (mm), mean ± SD	27.56 ± 19.24	34.17 ± 19.24	28.64 ± 19.24	< 0.001
No. of stents per target vessel, mean ± SD	1.06 ± 0.65	1.28 ± 0.65	1.23 ± 0.65	0.029
Post-stent non-compliant balloon use, n (%)	1970 (52%)	1335 (54%)	293 (52%)	0.301
Procedures with femoral artery approach, n (%)	3087 (81%)	1998 (81%)	450 (79%)	0.378
Vessels treated by major stent types, n (%)				
Xience family		995 (40%)		
Promus family		1143 (46%)		
Resolute family		978 (40%)		
Endeavor		162 (7%)		
Nobori			482 (85%)	
Biomatrix			185 (33%)	
Orsiro			22 (4%)	
Outcomes				
Cases of TVR, n (%)	222 (5.8%)	97 (3.9%)	18 (3.2%)	0.462
Observation period, days	730.31 ± 463.78	693.98 ± 463.78	724.12 ± 463.78	0.098

SD, standard deviation; DP-DES, durable polymer drug eluting stents; BP-DES, biodegradable polymer drug eluting stents; DAPT, dual antiplatelet therapy; LAD, left anterior descending artery; RCA, right coronary artery; LCx, left circumflex; LM, left main; TVR, target vessel revascularization.

*DP-DES group and BP-DES group were compared by Pearson’s chi-squared test for categorical variables and by Student’s *t*-test for continuous variables

### Validation of findings to demonstrate similar findings to previous studies

**Fig 2:**
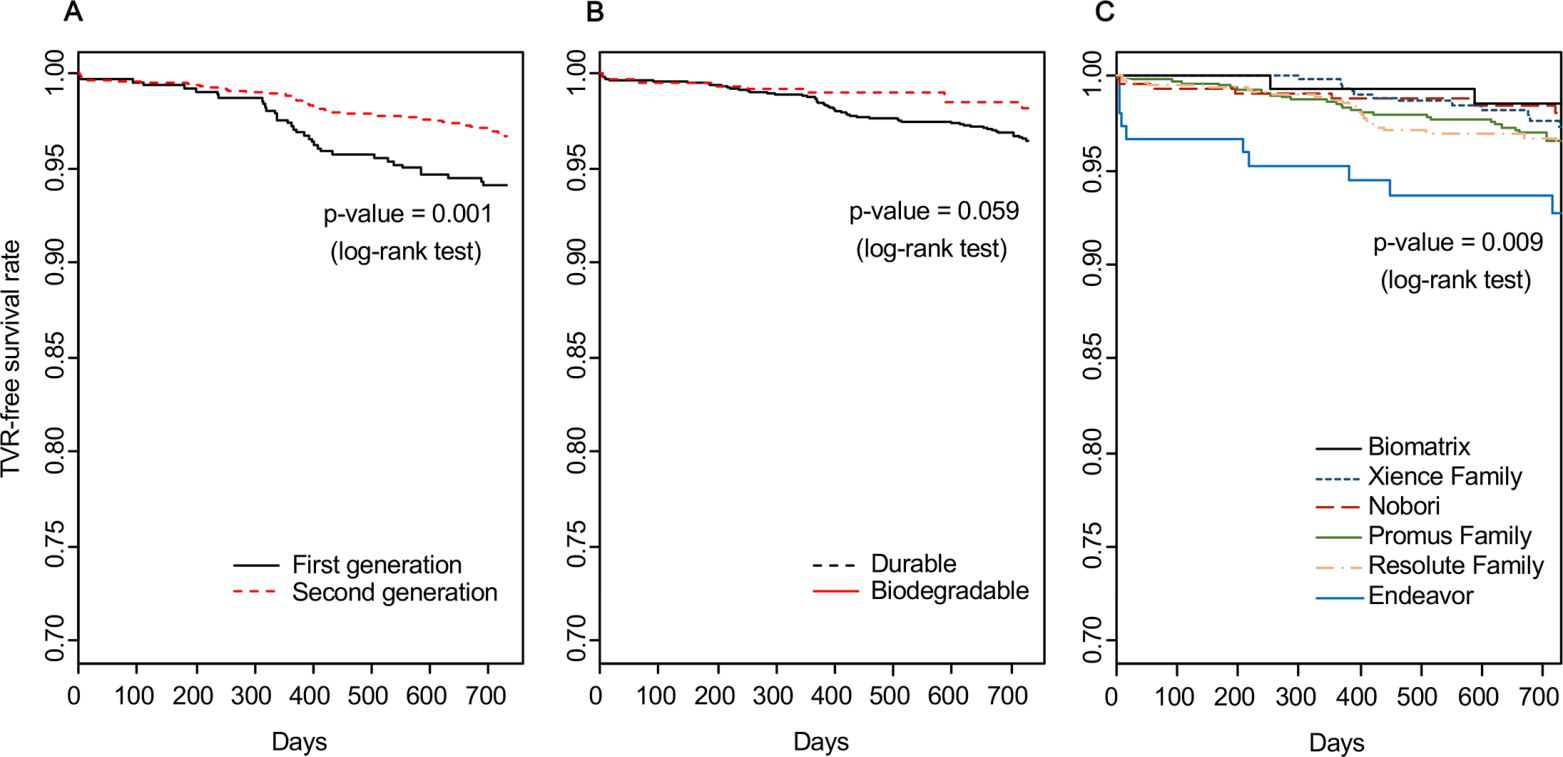
Comparison of target vessel revascularization free survival between the first- and second-generation stents.

To validate our data warehouse, we compared the TVR risk of the second-generation DES with that of the first-generation DES using Kaplan-Meier analysis and Cox proportional hazard regression. The TVR risk of second-generation stents was lower (adjusted HR: 0.423; 95% confidence interval (CI): 0.284–0.630), which was in accordance with previous studies (Fig 2A). The known predictors of TVR, including age, hypertension, diabetes mellitus, emergent PCI, and DAPT over 12 months, are also shown as significant (Table 4). These results indicate that the data in the constructed database are reliable and could be used for further analyses.

**Table 4:** Hazard ratios of first- versus second-generation stents and the observed variables in PCI patients.

Variable	HR (95% CI)	*p*-value
Second-generation stents (reference, first-generation stents)	0.423 (0.284–0.630)	< 0.001
Age	0.978 (0.962–0.994)	0.009
Sex (male)	1.010 (0.652–1.564)	0.966
Hypertension	3.469 (2.294–5.245)	< 0.001
Diabetes mellitus	2.100 (1.420–3.107)	< 0.001
Total stent length	1.005 (0.983–1.027)	0.661
Average stent diameter	0.943 (0.571–1.556)	0.818
Non-compliant high pressure balloon use	1.349 (0.919–1.980)	0.126
Target vessel (reference, LAD)		
LCx	0.697 (0.401–1.211)	0.200
LM	0.415 (0.163–1.055)	0.065
RCA	1.121 (0.732–1.716)	0.599
No. of stents used	0.833 (0.419–1.656)	0.601
Radial artery approach	0.714 (0.405–1.259)	0.244
Emergent PCI	3.270 (2.025–5.280)	< 0.001
DAPT over 12 months	0.389 (0.270–0.559)	< 0.001

HR, hazard ratio; CI, confidence interval; LAD, left anterior descending artery; RCA, right coronary artery; LCx, left circumflex; LM, left main; DAPT, dual antiplatelet therapy; PCI, percutaneous coronary intervention.

### New findings: BP-DES versus DP-DES

The baseline clinical characteristics of the BP-DES and DP-DES groups were similar (Table 3). Compared with the DP-DES group, RCA and LM were less frequently treated in the BP-DES group, and patients in the DP-DES group had a lower number of stents with smaller diameters and shorter lengths per target vessel. The rate of TVR was not significantly different between the BP-DES and DP-DES groups (3.2% vs. 3.9%, *p* = 0.462).

We compared the risk of TVR between the BP-DES and DP-DES groups. In patients treated with BP-DES, the TVR-free survival tended to be higher (log rank, *p* = 0.059) and the HR of TVR was lower without statistical significance (adjusted HR 0.568, 95% CI 0.281–1.147, *p* = 0.114) compared to patients treated with DP-DES (Table 5). However, in the Kaplan-Meier plot, the TVR-free survival rates of the two types of stents diverged at approximately 1 year after the index procedure was performed (Fig 2B).

**Table 5:** Hazard ratios of DP-DES vs. BP-DES and the observed variables in PCI patients.

Variable	HR (95% CI)	*p*-value
Biodegradable stents (reference, durable stents)	0.568 (0.281–1.147)	0.114
Age	0.980 (0.961–0.999)	0.040
Sex (male)	1.008 (0.597–1.702)	0.975
Hypertension	5.111 (2.983–8.757)	< 0.001
Diabetes mellitus	2.195 (1.386–3.477)	0.001
Total stent length	1.007 (0.982–1.032)	0.588
Average stent diameter	1.031 (0.577–1.843)	0.918
Non-compliant high pressure balloon use	1.392 (0.879–2.203)	0.158
Target vessel (reference, LAD)		
LCx	0.417 (0.196–0.887)	0.023
LM	0.319 (0.097–1.052)	0.061
RCA	0.967 (0.589–1.588)	0.894
No. of stents used	0.823 (0.381–1.774)	0.618
Radial artery approach	0.411 (0.178–0.951)	0.038
Emergent PCI	3.861 (2.273–6.559)	< 0.001
DAPT over 12 months	0.389 (0.251–0.603)	< 0.001

HR, hazard ratio; CI, confidence interval; LAD, left anterior descending artery; RCA, right coronary artery; LCx, left circumflex; LM, left main; DAPT, dual antiplatelet therapy; PCI, percutaneous coronary intervention.

### Within-class comparisons of DP-DES and BP-DES

Fig 2C shows the within-class comparison of TVR-free survival among the second-generation DES using Cox proportional hazard regression. The Endeavor stents showed a significantly higher HR (adjusted HR 2.576, 95% CI 1.273–5.210) than the other second-generation stents (Table 6). Other comparisons did not yield statistical significance, although BP-DES (i.e., Nobori and Biomatrix) showed a trend for lower HRs.

**Table 6:** Adjusted TVR risk of each stent compared to the other second generation stents.

Variable	Adjusted HR* (95%CI)	*p*-value
Xience family	0.717 (0.392–1.312)	0.280
Promus family	1.096 (0.691–1.740)	0.696
Resolute family	1.145 (0.697–1.880)	0.594
Endeavor	2.576 (1.273–5.210)	0.008
Nobori	0.721 (0.328–1.585)	0.416
Biomatrix	0.376 (0.091–1.552)	0.176

HR, hazard ratio; CI, confidence interval.

*Adjusted for age, sex, hypertension, diabetes mellitus, total length and average diameter of stents, non-compliant high pressure balloon use, target vessel (LAD, LCx, LM, or RCA), the number of stent used, approach artery, emergent percutaneous coronary intervention, and dual antiplatelet therapy over 12 months.

## Discussion

In this study, we extracted PCI-related parameters and constructed a PCI data warehouse from free-text electronic patient records with high accuracy. Using this data warehouse, we identified practice-based evidence to assess the performance of various coronary stents. In a self-validation analysis, the present study reaffirmed the established findings that newer generation DES are superior to first-generation DES in terms of TVR. Moreover, we provided clinically meaningful evidence that BP-DES were associated with a lower TVR risk than DP-DES, particularly 1 year after the index procedure. The Endeavor family of stents was significantly associated with higher risk of TVR among the newer generation stents.

Our findings support the results of previous studies and provide novel findings regarding the comparative efficacy of various newer types of DES in an unrestricted real-world clinical setting. A study based on the Swedish Coronary Angiography and Angioplasty Registry (SCAAR) showed similar results to this study when comparing older and newer generation DES [22]. Although the classification of Endeavor stents was different (old generation DES in the SCAAR study and second-generation DES in this study) the adjusted cumulative risk of target lesion revascularization (TLR) by PCI for up to 2 years (adjusted HR 0.60, 95% CI 0.51–0.70) was comparable to that of this study (adjusted HR 0.423, 95% CI 0.284–0.630). Moreover, when comparing each DES, the cumulative restenosis plot in the SCAAR study and the Kaplan-Meier plot in this study both showed that the TVR risk for Endeavor stents was higher than that of other stents [23]. Also, although there were no significant differences between the TVR HR of DP-DES and BP-DES for the 2-year outcome, the occurrence of TVR of the two stent types diverged after approximately 1 year. This reflects the results of the LEADERS trial, which showed a reduction in very late stent thrombosis (> 1 year) and a significant reduction in all-cause revascularization for BP-DES [24]. However, these results remain debatable, as some recent clinical trials state that the safety and efficacy of these stents did not differ [25, 26].

Previous studies have focused on comparing only a few types of stents; they were unable to make various comparisons due to the inherent limitations of clinical trials, which require numerous resources and are time-consuming. These studies were often confined to comparison of two or three types of stents and sometimes included only short follow-up periods (1 year), making it difficult to compare various stents from a single data source [27–29]. Also, as developments in DES technology occur rapidly, many studies become obsolete after just a few years, raising further questions regarding the timing and cost-effectiveness of prospective clinical trials.

In contrast, we were able to include a large number of procedures, with low cost and within a short period, by extracting information from reports stored in EHRs. We were also able to obtain data regarding the newer BP-DES. To do so, we developed text-mining tools using regular expression. Three different researchers developed mining programs independently and subsequently compared their results to ensure reliable data. Our data showed high validity and reliability when compared with the gold standard and already established researches. The sensitivity and specificity were 0.996 and 1.000, respectively, and the F-measure was 0.995. We confirmed that the second-generation DES are associated with significantly lower TVR risk than first-generation DES.

The high sensitivity and specificity we obtained are primarily the result of both the inherent characteristics of regular expression itself and the extensive reviews and revisions that we performed. High specificity was achieved because our expressions matched only the strings that were defined in the script, thus avoiding false positives. However, to ensure high sensitivity, it was paramount that all forms of abbreviations and spellings were pre-defined and taken into account. Although a pre-defined vessel and stent table was made, it was not sufficient to accommodate the various forms of the terms used. Therefore, the script review by the three programmers was of great importance to ensure high sensitivity. Using three individual scripts, it was possible to take into account the different forms other programmers had found, and an almost perfect match was obtained using the three scripts. The principal differences are the expressions used to match the patterns. A particular script may fail to extract certain words that other scripts extract, so these were revised during each round. This process was sufficient to reach a surprisingly high sensitivity.

The construction of a PCI data warehouse opens up possibilities for further research. First, our data warehouse contained information concerning the implemented stents and a detailed description of each stent in each target vessel (i.e., brand, number of stents, average diameter, and total length). Moreover, the analyses were strengthened using covariates (i.e., age, sex, hypertension, diabetes mellitus, and DAPT duration). Lastly, a total of 5,612 treated vessels from 3,817 PCI procedures were included, which resulted in a sample size large enough to achieve statistical validity.

The fact that EHRs stored solely for practice purposes can be used for research also opens up new possibilities. With proper extraction and organization, through application of technologies such as regular expression and natural language processing (NLP), the majority of EHRs could be used in a research setting. This study encourages the use of the vast data that are stored in the EHRs of hospitals, even those in free-text form.

There are several known NLP tools, which extract information through semantic analysis of medical records, such as the Linguistic Strings Project and the Medical Language Extraction and Encoding system (MedLEE) [30, 31]. However, most of these were developed for texts in English, while the majority of medical records in Korea are written in both English and Korean. There are also terms and abbreviations that are used only in Korea, making it difficult to apply existing tools directly to Korean medical records. However, the development of new NLP tools to analyze Korean EHRs would be expensive and labor-intensive. In addition, the CAG and PCI reports that we used did not contain complete sentences, but rather, were in a semi-structured format. Thus, pattern matching, not semantic analysis of the text, was the method of choice, particularly regular expression. When semi-structured medical records are written in both English and a local language (here, Korean), NLP tools cannot be used, and regular expression may be cost-effective and reliable when employed to extract information.

This study was not without limitations. First, the data warehouse was based solely upon procedures and prescriptions performed at Ajou University Hospital. Although patients are likely to return for follow-ups, some may choose different hospitals for further treatment. Others who were initially treated at another hospital (e.g., emergency reasons) may have subsequently visited Ajou University Hospital, possibly resulting in underreported outcomes and insufficient information on baseline characteristics. This is the reason why we only evaluated TVR rather than myocardial infarction and death, which are also major outcomes of PCI. The second limitation was that using regular expression may have caused unknown errors in cases with critical spelling mistakes on reports. Although most spelling mistakes were accounted for in our scripts, some extremely unique errors may have been missed, causing an imperfect sensitivity. Third, we only compared the list of pairs that consisted of the target vessel and stent categories to evaluate the degree of agreement between information retrieved using each script and the gold standard. Therefore, we could not determine how well the other variables were extracted other than the two mentioned above. However, it makes sure that the complete set of information was extracted, because if any of the three variables (stent name, diameter, and length) were missing, we determined that there was no record of a stent insertion in the corresponding vessel. The other variables (route of approach [radial or femoral], whether emergent PCI was performed or not, and whether a non-compliant high-pressure balloon was applied or not) were not evaluated in any way.

## Conclusions

A PCI data warehouse, constructed using regular expression reports on CAG and PCI stored in EHRs, was useful and reliable to evaluate the clinical performance of coronary stents. In terms of TVR, our study confirmed previous results demonstrating that second-generation DES are superior to first-generation DES, and demonstrated that Endeavor stents showed the highest event rate among the newer generation DES. BP-DES had a similar efficacy to DP-DES, but were associated with a superior long-term outcome after 1 year of observation. It is encouraging for further research that free-text reports originally stored for diagnostic purposes can be processed into forms that are appropriate for research.

## Supporting information

S1 File Demonstration of the regular expression script. (DOCX)

S2 File A Sample data set of the PCI data warehouse. (XLSX)

## References

[1] Nabel EG, Braunwald E. A tale of coronary artery disease and myocardial infarction. The New England journal of medicine. 2012;366(1):54–63. doi: 10.1056/NEJMra1112570 . 22216842 10.1056/NEJMra1112570

[2] Mozaffarian D, Benjamin EJ, Go AS, Arnett DK, Blaha MJ, Cushman M, et al Heart disease and stroke statistics—2015 update: a report from the American Heart Association. Circulation. 2015;131(4):e29–322. doi: 10.1161/CIR.0000000000000152 . 25520374 10.1161/CIR.0000000000000152

[3] Sigwart U, Puel J, Mirkovitch V, Joffre F, Kappenberger L. Intravascular stents to prevent occlusion and restenosis after transluminal angioplasty. The New England journal of medicine. 1987;316(12):701–6. doi: 10.1056/NEJM198703193161201 . 2950322 10.1056/NEJM198703193161201

[4] Serruys PW, Unger F, Sousa JE, Jatene A, Bonnier HJ, Schonberger JP, et al Comparison of coronary-artery bypass surgery and stenting for the treatment of multivessel disease. The New England journal of medicine. 2001;344(15):1117–24. doi: 10.1056/NEJM200104123441502 . 11297702 10.1056/NEJM200104123441502

[5] Spaulding C, Henry P, Teiger E, Beatt K, Bramucci E, Carrie D, et al Sirolimus-eluting versus uncoated stents in acute myocardial infarction. The New England journal of medicine. 2006;355(11):1093–104. doi: 10.1056/NEJMoa062006 . 16971716 10.1056/NEJMoa062006

[6] Stone GW, Moses JW, Ellis SG, Schofer J, Dawkins KD, Morice MC, et al Safety and efficacy of sirolimus- and paclitaxel-eluting coronary stents. The New England journal of medicine. 2007;356(10):998–1008. doi: 10.1056/NEJMoa067193 . 17296824 10.1056/NEJMoa067193

[7] Nakazawa G, Finn AV, Joner M, Ladich E, Kutys R, Mont EK, et al Delayed arterial healing and increased late stent thrombosis at culprit sites after drug-eluting stent placement for acute myocardial infarction patients: an autopsy study. Circulation. 2008;118(11):1138–45. doi: 10.1161/CIRCULATIONAHA.107.762047 . 18725485 10.1161/CIRCULATIONAHA.107.762047

[8] Canan T, Lee MS. Drug-eluting stent fracture: incidence, contributing factors, and clinical implications. Catheterization and cardiovascular interventions: official journal of the Society for Cardiac Angiography & Interventions. 2010;75(2):237–45. doi: 10.1002/ccd.22212 . 20025045 10.1002/ccd.22212

[9] Chakravarty T, White AJ, Buch M, Naik H, Doctor N, Schapira J, et al Meta-analysis of incidence, clinical characteristics and implications of stent fracture. The American journal of cardiology. 2010;106(8):1075–80. doi: 10.1016/j.amjcard.2010.06.010 . 20920641 10.1016/j.amjcard.2010.06.010

[10] Shaikh F, Maddikunta R, Djelmami-Hani M, Solis J, Allaqaband S, Bajwa T. Stent fracture, an incidental finding or a significant marker of clinical in-stent restenosis? Catheterization and cardiovascular interventions: official journal of the Society for Cardiac Angiography & Interventions. 2008;71(5):614–8. doi: 10.1002/ccd.21371 . 18360853 10.1002/ccd.21371

[11] Kaul U, Bangalore S, Seth A, Arambam P, Abhaychand RK, Patel TM, et al Paclitaxel-Eluting versus Everolimus-Eluting Coronary Stents in Diabetes. The New England journal of medicine. 2015;373(18):1709–19. doi: 10.1056/NEJMoa1510188 . 26466202 10.1056/NEJMoa1510188

[12] Kobayashi N, Ito Y, Nakano M, Araki M, Hirano K, Yamawaki M, et al Incidence and Characteristics of Late Catch-Up Phenomenon Between Sirolimus-Eluting Stent and Everolimus-Eluting Stent: A Propensity Matched Study. Journal of interventional cardiology. 2015 doi: 10.1111/joic.12247 . 26487339 10.1111/joic.12247

[13] Colombo A, Karvouni E. Biodegradable stents: "fulfilling the mission and stepping away". Circulation. 2000;102(4):371–3. . 10908206 10.1161/01.cir.102.4.371

[14] Stefanini GG, Byrne RA, Serruys PW, de Waha A, Meier B, Massberg S, et al Biodegradable polymer drug-eluting stents reduce the risk of stent thrombosis at 4 years in patients undergoing percutaneous coronary intervention: a pooled analysis of individual patient data from the ISAR-TEST 3, ISAR-TEST 4, and LEADERS randomized trials. European heart journal. 2012;33(10):1214–22. doi: 10.1093/eurheartj/ehs086 . 22447805 10.1093/eurheartj/ehs086

[15] Stefanini GG, Kalesan B, Serruys PW, Heg D, Buszman P, Linke A, et al Long-term clinical outcomes of biodegradable polymer biolimus-eluting stents versus durable polymer sirolimus-eluting stents in patients with coronary artery disease (LEADERS): 4 year follow-up of a randomised non-inferiority trial. Lancet. 2011;378(9807):1940–8. doi: 10.1016/S0140-6736(11)61672-3 . 22075451 10.1016/S0140-6736(11)61672-3

[16] Bartorelli AL, Serruys PW, Miquel-Hebert K, Yu S, Pierson W, Stone GW, et al An everolimus-eluting stent versus a paclitaxel-eluting stent in small vessel coronary artery disease: a pooled analysis from the SPIRIT II and SPIRIT III trials. Catheterization and cardiovascular interventions: official journal of the Society for Cardiac Angiography & Interventions. 2010;76(1):60–6. doi: 10.1002/ccd.22452 . 20578194 10.1002/ccd.22452

[17] Stefanini GG, Holmes DR Jr. Drug-eluting coronary-artery stents. The New England journal of medicine. 2013;368(3):254–65. doi: 10.1056/NEJMra1210816 . 23323902 10.1056/NEJMra1210816

[18] Task Force M, Montalescot G, Sechtem U, Achenbach S, Andreotti F, Arden C, et al 2013 ESC guidelines on the management of stable coronary artery disease: the Task Force on the management of stable coronary artery disease of the European Society of Cardiology. European heart journal. 2013;34(38):2949–3003. doi: 10.1093/eurheartj/eht296 . 23996286 10.1093/eurheartj/eht296

[19] Kaur G. USAGE OF REGULAR EXPRESSIONS IN NLP. International Journal of Research in Engineering and Technology IJERT. 2014;03(01):7.

[20] Wickham H. stringr: Simple, Consistent Wrappers for Common String Operations 2015. Available from: http://CRAN.R-project.org/package=stringr.

[21] Kho AN, Hayes MG, Rasmussen-Torvik L, Pacheco JA, Thompson WK, Armstrong LL, et al Use of diverse electronic medical record systems to identify genetic risk for type 2 diabetes within a genome-wide association study. J Am Med Inform Assoc. 2012;19(2):212–8. doi: 10.1136/amiajnl-2011-000439 ; PubMed Central PMCID: PMCPMC3277617. 22101970 10.1136/amiajnl-2011-000439 PMC3277617

[22] Sarno G, Lagerqvist B, Frobert O, Nilsson J, Olivecrona G, Omerovic E, et al Lower risk of stent thrombosis and restenosis with unrestricted use of 'new-generation' drug-eluting stents: a report from the nationwide Swedish Coronary Angiography and Angioplasty Registry (SCAAR). European heart journal. 2012;33(5):606–13. doi: 10.1093/eurheartj/ehr479 . 22232428 10.1093/eurheartj/ehr479

[23] Sarno G, Lagerqvist B, Carlsson J, Olivecrona G, Nilsson J, Calais F, et al Initial clinical experience with an everolimus eluting platinum chromium stent (Promus Element) in unselected patients from the Swedish Coronary Angiography and Angioplasty Registry (SCAAR). International journal of cardiology. 2013;167(1):146–50. doi: 10.1016/j.ijcard.2011.12.057 . 22244480 10.1016/j.ijcard.2011.12.057

[24] Serruys PW, Farooq V, Kalesan B, de Vries T, Buszman P, Linke A, et al Improved safety and reduction in stent thrombosis associated with biodegradable polymer-based biolimus-eluting stents versus durable polymer-based sirolimus-eluting stents in patients with coronary artery disease: final 5-year report of the LEADERS (Limus Eluted From A Durable Versus ERodable Stent Coating) randomized, noninferiority trial. JACC Cardiovascular interventions. 2013;6(8):777–89. doi: 10.1016/j.jcin.2013.04.011 . 23968698 10.1016/j.jcin.2013.04.011

[25] Kaiser C, Galatius S, Jeger R, Gilgen N, Skov Jensen J, Naber C, et al Long-term efficacy and safety of biodegradable-polymer biolimus-eluting stents: main results of the Basel Stent Kosten-Effektivitats Trial-PROspective Validation Examination II (BASKET-PROVE II), a randomized, controlled noninferiority 2-year outcome trial. Circulation. 2015;131(1):74–81. doi: 10.1161/CIRCULATIONAHA.114.013520 . 25411159 10.1161/CIRCULATIONAHA.114.013520

[26] Vlachojannis GJ, Smits PC, Hofma SH, Togni M, Vazquez N, Valdes M, et al Long-term clinical outcomes of biodegradable polymer biolimus-eluting stents versus durable polymer everolimus-eluting stents in patients with coronary artery disease: three-year follow-up of the COMPARE II (Abluminal biodegradable polymer biolimus-eluting stent versus durable polymer everolimus-eluting stent) trial. EuroIntervention: journal of EuroPCR in collaboration with the Working Group on Interventional Cardiology of the European Society of Cardiology. 2015;11(3):272–9. doi: 10.4244/EIJV11I3A53 . 26196753 10.4244/EIJV11I3A53

[27] Stone GW, Rizvi A, Newman W, Mastali K, Wang JC, Caputo R, et al Everolimus-eluting versus paclitaxel-eluting stents in coronary artery disease. The New England journal of medicine. 2010;362(18):1663–74. doi: 10.1056/NEJMoa0910496 . 20445180 10.1056/NEJMoa0910496

[28] Sen H, Lam MK, Lowik MM, Danse PW, Jessurun GA, van Houwelingen KG, et al Clinical Events and Patient-Reported Chest Pain in All-Comers Treated With Resolute Integrity and Promus Element Stents: 2-Year Follow-Up of the DUTCH PEERS (DUrable Polymer-Based STent CHallenge of Promus ElemEnt Versus ReSolute Integrity) Randomized Trial (TWENTE II). JACC Cardiovascular interventions. 2015;8(7):889–99. doi: 10.1016/j.jcin.2015.01.033 . 26003019 10.1016/j.jcin.2015.01.033

[29] Kedhi E, Joesoef KS, McFadden E, Wassing J, van Mieghem C, Goedhart D, et al Second-generation everolimus-eluting and paclitaxel-eluting stents in real-life practice (COMPARE): a randomised trial. Lancet. 2010;375(9710):201–9. doi: 10.1016/S0140-6736(09)62127-9 . 20060578 10.1016/S0140-6736(09)62127-9

[30] Sager N, Friedman C., Chi E., et al The analysis and processing of clinical narrative. Medinfo. Amsterdam (Holland): Elsevier; 1986 p. 1101–5.

[31] Friedman C, Johnson SB, Forman B, Starren J. Architectural requirements for a multipurpose natural language processor in the clinical environment. Proceedings / the Annual Symposium on Computer Application [sic] in Medical Care Symposium on Computer Applications in Medical Care. 1995:347–51. ; PubMed Central PMCID: PMC2579112. 8563299 PMC2579112

