# Supplementary material for: Extracting information from free-text electronic patient records to identify practice-based evidence of the performance of coronary stents
Source: PLoS One. 2017 Aug 11;12(8):e0182889. doi: 10.1371/journal.pone.0182889 (PMC5553787; doi:10.1371/journal.pone.0182889)
Supplement: S1 File — (DOCX) [file pone.0182889.s001.docx]

Supporting Information

Title: Extracting information from free-text electronic patient records to identify practice-based evidence of the performance of coronary stents

Yoon Seob Kim, Dukyong Yoon, JungHyun Byun, Hojun Park, Ahram Lee, Il Hyun Kim, Sukhoon Lee, Hong-Seok Lim, Rae Woong Park

A sample of the coronary angiography report

Text<-" Diagnostic CAG/r/n/r/n/r/n 1. Rt. Femoral artery approach/r/n/r/n/r/n 2. JL 6/4F and JR 6/3.5F catheters were used/r/n/r/n/r/n 3. Findings/r/n/r/n/r/n 1) LM: normal/r/n/r/n/r/n 2) LAD: pLAD: tubular eccentric 48%/r/n/r/n/r/n mLAD: No ISR at previous stented site/r/n/r/n/r/n distal edge: tubular concentric 52%/r/n/r/n/r/n 3) LCX: p-dLCX: diffuse irregular up to 68%/r/n/r/n/r/n (small vessel)/r/n/r/n/r/n OM: total occlusion /r/n/r/n/r/n (TIMI 0, small vessel)/r/n/r/n/r/n 4) RCA: p-mRCA: diffuse irregular up to 55%/r/n/r/n/r/n dRCA: diffuse irregular up to 39%/r/n/r/n/r/n/r/n/r/n/r/n *Diagnostic angiography 시행 후 acute onset/r/n/r/n/r/n chest pain 및 ECG상 ST elevation 소견 보여 /r/n/r/n/r/n angiography 다시 시행함 /r/n/r/n/r/n -> proximal edge에 total occluion 소견 보임/r/n/r/n/r/n (TIMI 0, TMPG 0)/r/n/r/n/r/n/r/n/r/n/r/nPCI/r/n/r/n/r/n 1.EBU 7/3.5F guiding catheter was used. /r/n/r/n/r/n 2.Primary PCI was performed./r/n/r/n/r/n 3.PCI Descriptions/r/n/r/n/r/n mLAD: predilation with Lacrosse 2.5/20mm(6 atm)/r/n/r/n/r/n -> total occlusion 소견 여전히 보임/r/n/r/n/r/n -> suction with Thrombuster catether/r/n/r/n/r/n (aspiration material: red clot)/r/n/r/n/r/n -> distal edge-dLAD에 diffuse stenotic /r/n/r/n/r/n lesion 보임 (TIMI 1, TMPG 0)/r/n/r/n/r/n distal edge-dLAD:/r/n/r/n/r/n Resolute integrity 2.75/26mm(9 atm)/r/n/r/n/r/n (*spastic component가 동반된 것으로 판단되어/r/n/r/n/r/n stent diameter를 작은 것으로 삽입,/r/n/r/n/r/n overlapped with previous stent)/r/n/r/n/r/n -> slow flow was seen (TIMI 2, TMPG 1)/r/n/r/n/r/n -> IC adenosine 후 호전/r/n/r/n/r/n -> Final flow TIMI 3, TMPG 2/r/n/r/n/r/n -> far distal LAD에 intraluminal filling/r/n/r/n/r/n defect (R/O thrombus) 소견 보임 /r/n/r/n/r/n far dLAD: PTCA with miniTrek 1.5/15mm(12 atm)/r/n/r/n/r/n/r/n/r/n/r/n OM: PTCA with miniTrek 1.5/15mm(10 atm)/r/n/r/n/r/n -> final flow TIMI 3, TMPG 1/r/n/r/n/r/n 4.Successful PCI without complications/r/n/r/n/r/n

"

Extraction of the "PCI" component of the report

library(stringr)
PCI<-str_extract(Text,"PCI [Dd]e.*")
PCI<-gsub("(/r/n *)|(/r/n( */r/n)*)","/r/n ",PCI)
print(PCI)

## [1] "PCI Descriptions/r/n mLAD: predilation with Lacrosse 2.5/20mm(6 atm)/r/n -> total occlusion 소견 여전히 보임/r/n -> suction with Thrombuster catether/r/n (aspiration material: red clot)/r/n -> distal edge-dLAD에 diffuse stenotic /r/n lesion 보임 (TIMI 1, TMPG 0)/r/n distal edge-dLAD:/r/n Resolute integrity 2.75/26mm(9 atm)/r/n (*spastic component가 동반된 것으로 판단되어/r/n stent diameter를 작은 것으로 삽입,/r/n overlapped with previous stent)/r/n -> slow flow was seen (TIMI 2, TMPG 1)/r/n -> IC adenosine 후 호전/r/n -> Final flow TIMI 3, TMPG 2/r/n -> far distal LAD에 intraluminal filling/r/n defect (R/O thrombus) 소견 보임 /r/n far dLAD: PTCA with miniTrek 1.5/15mm(12 atm)/r/n OM: PTCA with miniTrek 1.5/15mm(10 atm)/r/n -> final flow TIMI 3, TMPG 1/r/n 4.Successful PCI without complications/r/n "

(Step 1) Extracting all words between "new-line (\n)" and ":" in order to find the target vessels of the Percutaneous Coronary Intervention

tv.loc<-list()
tv2<-list()
for(x in 1:length(PCI)){

 if(is.na(PCI[[x]])==T){
 tv.loc<-append(tv.loc,list(NULL))
 tv2<-append(tv2,list(NA))
 }

 if(is.na(PCI[[x]])==F){
 tv<-rbind(c(0,0),str_locate_all(PCI[[x]],
 "(LM.{0,5}LAD|LM.{0,5}LCx|Ramus|RI|Dx|LAD|L[Cc][Xx]|RCA|PDA|PLV|LM|OM|D1|D2|PLB|[Dd]iagonal)(.{0,8}[:;])")[[1]])
 if(nrow(tv)!=1){
 tv2<-append(tv2,list(sapply(2:nrow(tv),function(y)substr(PCI[[x]],tv[y,1],tv[y,2]))))
 tv.loc<-append(tv.loc,str_locate_all(PCI[[x]],"(LM.{0,5}LAD|LM.{0,5}LCx|Ramus|RI|Dx|LAD|L[Cc][Xx]|RCA|PDA|PLV|LM|OM|D1|D2|PLB|[Dd]iagonal)(.{0,8}[:;])"))
 }
 if(nrow(tv)==1){
 tv2<-append(tv2,NA)
 tv.loc<-append(tv.loc,list(NULL))
 }
 }
}
tv3<-unlist(tv2)[complete.cases(unlist(tv2))]
print(tv3)

## [1] "LAD:" "LAD:" "LAD:" "OM:"

(Step 2) Matching the extracted words with pre-defined vessel categories

lesions<-tv3
RCA<-grepl("PDA|PLB|PLV|RCA",lesions)
LM<-grepl("LM",lesions)
LAD<-grepl("LAD|D[12xX]|[Dd]?iagonal",lesions)
LCx<-grepl("OM|RI|OM1|OM2|Ramus|Raus|LCX|LCx|Lcx",lesions)


lesions[RCA]<-"RCA"
lesions[LCx&!LM]<-"LCx"
lesions[LAD&!LM]<-"LAD"
lesions[LAD&LM]<-"LM-LAD"
lesions[LCx&LM]<-"LM-LCx"
lesions[LM&!LCx&!LAD]<-"LM"
print(lesions)

## [1] "LAD" "LAD" "LAD" "LCx"

(Step 3) Extracting all words between the detected vessel names or between the detected vessel name and the end of report

str.loc<-tv.loc
good<-lapply(str.loc,function(x)length(x)>0)
str.loc[unlist(good)]<-lapply(str.loc[unlist(good)],invert_match)
str.ext<-list()
for(i in 1:length(str.loc)){
 if(length(str.loc[[i]])==0){
 str.ext<-append(str.ext,list(NULL))
 }
 if(length(str.loc[[i]])!=0){
 map<-str.loc[[i]]
 map[nrow(map),2]<-10000
 str.ext<-append(str.ext,list(sapply(1:nrow(map),function(y)substr(PCI[i],map[y,1],map[y,2]))[-1]))
 }
}
lstring.N<-str.ext

lengths.N<-sapply(str.ext,length)

strings<-unlist(lstring.N)
print(strings)

## [1] : " predilation with Lacrosse 2.5/20mm(6 atm)/r/n -> total occlusion 소견 여전히 보임/r/n -> suction with Thrombuster catether/r/n (aspiration material: red clot)/r/n -> distal edge-dLAD에 diffuse stenotic /r/n lesion 보임 (TIMI 1, TMPG 0)/r/n distal edge-d"

## [2] "/r/n Resolute integrity 2.75/26mm(9 atm)/r/n (*spastic component가 동반된 것으로 판단되어/r/n stent diameter를 작은 것으로 삽입,/r/n overlapped with previous stent)/r/n -> slow flow was seen (TIMI 2, TMPG 1)/r/n -> IC adenosine 후 호전/r/n -> Final flow TIMI 3, TMPG 2/r/n -> far distal LAD에 intraluminal filling/r/n defect (R/O thrombus) 소견 보임 /r/n far d"

## [3] " PTCA with miniTrek 1.5/15mm(12 atm)/r/n "

## [4] " PTCA with miniTrek 1.5/15mm(10 atm)/r/n -> final flow TIMI 3, TMPG 1/r/n 4.Successful PCI without complications/r/n "

Therefore, we get

split.data<-data.frame(lesions,strings)
colnames(split.data)<-c("Target_Vessel","Description")
print(split.data)

## Target_Vessel
## 1 LAD
## 2 LAD
## 3 LAD
## 4 LCx
## Description
## 1 predilation with Lacrosse 2.5/20mm(6 atm)/r/n -> total occlusion 소견 여전히 보임 /r/n -> suction with Thrombuster catether/r/n (aspiration material: red clot)/r/n -> distal edge-dLAD 에 diffuse stenotic /r/n lesion 보임 (TIMI 1, TMPG 0)/r/n distal edge-d
## 2 /r/n Resolute integrity 2.75/26mm(9 atm)/r/n (*spastic component가 동반된 것으로 판단되어/r/n stent diameter를 작은 것으로 삽입,/r/n overlapped with previous stent)/r/n -> slow flow was seen (TIMI 2, TMPG 1)/r/n -> IC adenosine 후 호전/r/n -> Final flow TIMI 3, TMPG 2/r/n -> far distal LAD에 intraluminal filling/r/n defect (R/O thrombus) 소견 보임 /r/n far d
## 3 PTCA with miniTrek 1.5/15mm(12 atm)/r/n
## 4 PTCA with miniTrek 1.5/15mm(10 atm)/r/n -> final flow TIMI 3, TMPG 1/r/n 4.Successful PCI without complications/r/n

The terms that come before "mm"

unlist(str_extract_all(PCI,"([[:alnum:]]* ){3}[[:digit:]]\\.[[:digit:]]{0,2}/[[:digit:]]{2}mm"))

## [1] "predilation with Lacrosse 2.5/20mm"
## [2] " Resolute integrity 2.75/26mm"
## [3] "PTCA with miniTrek 1.5/15mm"
## [4] "PTCA with miniTrek 1.5/15mm"

Among the extracted terms, "Resolute Integrity" is a word that refers to a stent.

The rest are terms that are used to indicate balloons that were used in the procedure

*In the original research, this was done on the entire data, and recurring phrases were reviewed by a cardiologist.

(Step 4) Matching the extracted words with pre-defined stent names

coroflex.isar<-"[Cc]oroflex [Ii][Ss][Aa][Rr]"
desyne<-"[Dd]esyne"
osiro<-"[Oo]siro|[Oo]rsiro"
vision<-"[Vv]ision"
zeta<-"[Zz]eta"
coroflex.blue<-"[Cc]oro[Ff]l?ex ?[Bb]lue|Cofoflex Blue|Corofelx blue"
driver<-"[Dd]river"
genoss<-"[Gg]enoss|GENOSS"
resolute.integrity<-"[Rr]esolute [Ii]ntegrity|[Rr]\\. ?[Ii]ntegrity|[Rr]esolutei [Ii]ntegrity|[Rr]esolute ?\\.[Ii]ntegrity|[Rr]esolute [^iI]|[Ii]ntegrity|[Rr]esolute intergrity|[Rr]esolute [Ii]|Resolute intetrity|Resolute integriyt"
biomatrix<-"[Bb]io [Mm]atrix|[Bb][Ii]o[Mm]atrix"
coroflex.please<-"[Cc]oroflex [Pp]lease"
xience<-"[Xx]ience [^XxPp]|[Xx]ience [Xx]per?dition|[^e] ?[Pp]rime|[Xx]ience [Pp]ri[mn]e|[Xx]pedition|X\\. ?[Pp]rime|priem|Xience Premier"
cypher<-"[Cc]ypher"
nobori<-"[Nn]obori|[Nn]ovori?|[Mm]obori"
endeavor<-"[Ee]ndeave|[Dd]ndeavor|[Xx]ndeavor|[Ee]ndeavor [Ii]ntegrity|[Ee][Nn]deavor"
taxus<-"[Tt]axus"
novolimus<-"[Nn]ovolimus"
p.element<-"P\\.element|([Pp]\\. ?[Ee]lem)|([Pp]romus [Ee])|([Pp]\\.[Ee])|[Pp]\\>[Ee]lement|[Ee]lement|[Pp]romus {1,2}[Pp]remier|[Pp].premier|Promus [^Ee]"
genous<-"[Gg]enous"
names<-c(coroflex.isar,desyne,osiro,vision,zeta,coroflex.blue,driver,genoss,resolute.integrity,biomatrix,coroflex.please,xience,cypher,nobori,endeavor,taxus,novolimus,p.element,genous)

stent.all<-NULL
for(i in 1:length(strings)){
 new.l<-str_extract_all(strings[i],names)
 stent.all<-c(stent.all,list(new.l))
}

stent_temp<-lapply(stent.all,function(x)sapply(x,length))
stent<-data.frame(matrix(unlist(stent_temp),ncol=19,byrow=T))
colnames(stent)<-c("coroflex.isar","desyne","osiro","vision","zeta","coroflex.blue","driver","genoss","resolute.integrity","biomatrix","coroflex.please","xience","cypher","nobori","endeavor","taxus","novolimus","p.element","genous")
print(stent)

## coroflex.isar desyne osiro vision zeta coroflex.blue driver genoss
## 1 0 0 0 0 0 0 0 0
## 2 0 0 0 0 0 0 0 0
## 3 0 0 0 0 0 0 0 0
## 4 0 0 0 0 0 0 0 0
## resolute.integrity biomatrix coroflex.please xience cypher nobori
## 1 0 0 0 0 0 0
## 2 1 0 0 0 0 0
## 3 0 0 0 0 0 0
## 4 0 0 0 0 0 0
## endeavor taxus novolimus p.element genous
## 1 0 0 0 0 0
## 2 0 0 0 0 0
## 3 0 0 0 0 0
## 4 0 0 0 0 0

(Step 5) Extracting the diameter and length information following the detected stent names (two numbers followed by "mm", which are separated by "/").

stent.info.l<-NULL
for(i in 1:length(strings)){
 new.l<-str_extract_all(strings[i],paste0("(",names,")"," *?(/r/n){0,3}","(.{0,8}[[:digit:]]{0,1}\\.[[:digit:]]{1,2}.{1,2}[[:digit:]]{1,2})",
 "(@(.{0,8}[[:digit:]]{0,1}\\.[[:digit:]]{1,2}.{1,2}[[:digit:]]{1,2}))?"))
 stent.info.l<-c(stent.info.l,list(new.l))
}

stent.info<-lapply(stent.info.l,unlist)
stent.info2<-lapply(stent.info,function(x)str_extract(x,"[[:digit:]].*"))
stent.info2[sapply(stent.info2,length)==0]<-0
s.diameter<-unlist(lapply(stent.info2,function(x)str_extract(x,"^[[:digit:]]\\.[[:digit:]]{1,2}")))
s.length<-unlist(lapply(stent.info2,function(x)str_extract(x,"[[:digit:]]{2}$")))
s.diameter[is.na(s.diameter)==1]<-0
s.length[is.na(s.length)==1]<-0
info<-data.frame(as.numeric(s.diameter),as.numeric(s.length))
colnames(info)<-c("Diameter","Length")
print(info)

## Diameter Length
## 1 0.00 0
## 2 2.75 26
## 3 0.00 0
## 4 0.00 0

(Step 6) Extracting information whether non-compliant high-pressure balloon was applied (whether the word "HP" or the name of the balloon was written) or not.

lstring3<-unlist(lstring.N)
HP<-as.numeric(grepl("[Hh][Pp]|[Pp]o?w?.{0,6}[Ll]acrosse|[Ff]ortis|[Dd]urastar|[Qq]uantum|[Vv]oyager",lstring3))
print(HP)

## [1] 0 0 0 0

The code above extracts the following information:

data<-data.frame(lesions,stent,info,HP)
data2<-aggregate(data[c(2:ncol(data))],by=list(Target_Vessel=data$lesions),sum)
data2[data2$Diameter>0,"Diameter"]<-data2[data2$Diameter>0,"Diameter"]/c(rowSums(data2[data2$Diameter>0,2:(ncol(data2)-3)]))
print(data2)

## Target_Vessel coroflex.isar desyne osiro vision zeta coroflex.blue
## 1 LAD 0 0 0 0 0 0
## 2 LCx 0 0 0 0 0 0
## driver genoss resolute.integrity biomatrix coroflex.please xience cypher
## 1 0 0 1 0 0 0 0
## 2 0 0 0 0 0 0 0
## nobori endeavor taxus novolimus p.element genous Diameter Length HP
## 1 0 0 0 0 0 0 2.75 26 0
## 2 0 0 0 0 0 0 0.00 0 0
